# Dissociated optic nerve fiber layer-like appearance indicating an internal limiting membrane defect associated with an epiretinal membrane: two case reports

**DOI:** 10.1186/s12886-022-02388-w

**Published:** 2022-04-14

**Authors:** Yuichiro Ishida, Naomichi Ota, Kotaro Tsuboi, Motohiro Kamei

**Affiliations:** Department of Ophthalmology, Aichi Medical University, Nagakute, 1-1, Yazako-karimata, Nagakute, Aichi 480-1195 Japan

**Keywords:** Dissociated optic nerve fiber layer-like appearance, Internal limiting membrane defect, Epiretinal membrane, Case report

## Abstract

**Background:**

We report for the first time a way to predict the 2-dimensional extension of an internal limiting membrane (ILM) defect by detecting the area with dissociated optic nerve fiber layer (DONFL)-like spots in the preoperative optical coherence tomography (OCT) en-face images.

**Case presentations:**

Case 1 was a 67-year-old man with metamorphopsia and decreased vision in his right eye. His best-corrected visual acuity (BCVA) was 20/100, with a pterygium, a moderate nuclear cataract, and an epiretinal membrane (ERM). Case 2 was a 73-year-old man with metamorphopsia and decreased vision in his left eye. His BCVA was 20/25, with a moderate nuclear cataract and an ERM. Both patients underwent simultaneous cataract surgery and pars plana vitrectomy with ERM and ILM peeling. Brilliant Blue G staining, performed before ERM and ILM peeling, revealed an unstained area. A careful evaluation of the area showed that it was not covered by either the ERM or ILM. A postoperative evaluation of the preoperative OCT images obtained from these cases showed DONFL-like low-brightness spots in the ILM defect area on the OCT en-face images.

**Conclusions:**

OCT en-face images may indicate the area of the ILM defect. To avoid iatrogenic damage to the retinal nerve fiber layer by touching/pinching it with forceps, detecting areas with DONFL-like spots in the preoperative OCT en-face images may be useful to predict an ILM defect.

## Background

Pars plana vitrectomy (PPV) with membrane peeling is performed to treat a symptomatic epiretinal membrane (ERM). Some surgeons also perform internal limiting membrane (ILM) peeling, which can damage the inner retinal layers and the physiology of the Müller cells, because the ILM is comprised of the basal membrane and the end-feet of the Müller cells [1, 2]. However, since the visual outcomes are similar between cases with/without ILM peeling and the ERM recurrence rate is lower in eyes with ILM peeling [3], this technique has become popular in ERM surgery.

When performing ILM peeling, it is common to use stains such as indocyanine green, trypan blue, or Brilliant Blue G (BBG) [4–6]. ERMs are thought to be present in areas that remain unstained. However, we sometimes encounter an area that is untouched but not stained with a dye and appears to have no ERM during membrane peeling. An ILM may not be present and fuzzy tissue may be present in the area. Touching the area or grasping the fuzzy tissue is dangerous because of the potential of rubbing/pinching the retinal nerve fiber layer (RNFL). Therefore, it is important to identify the area preoperatively.

We present two cases in which areas with dissociated optic nerve fiber layer (DONFL)-like low-brightness spots were detected on the optical coherence tomography (OCT) en-face images and a defect of the ILM in the area was confirmed during the ERM surgery. Both patients provided written informed consent for publication of patient-related materials.

## Case presentations

### Case 1

**Fig. 1:**
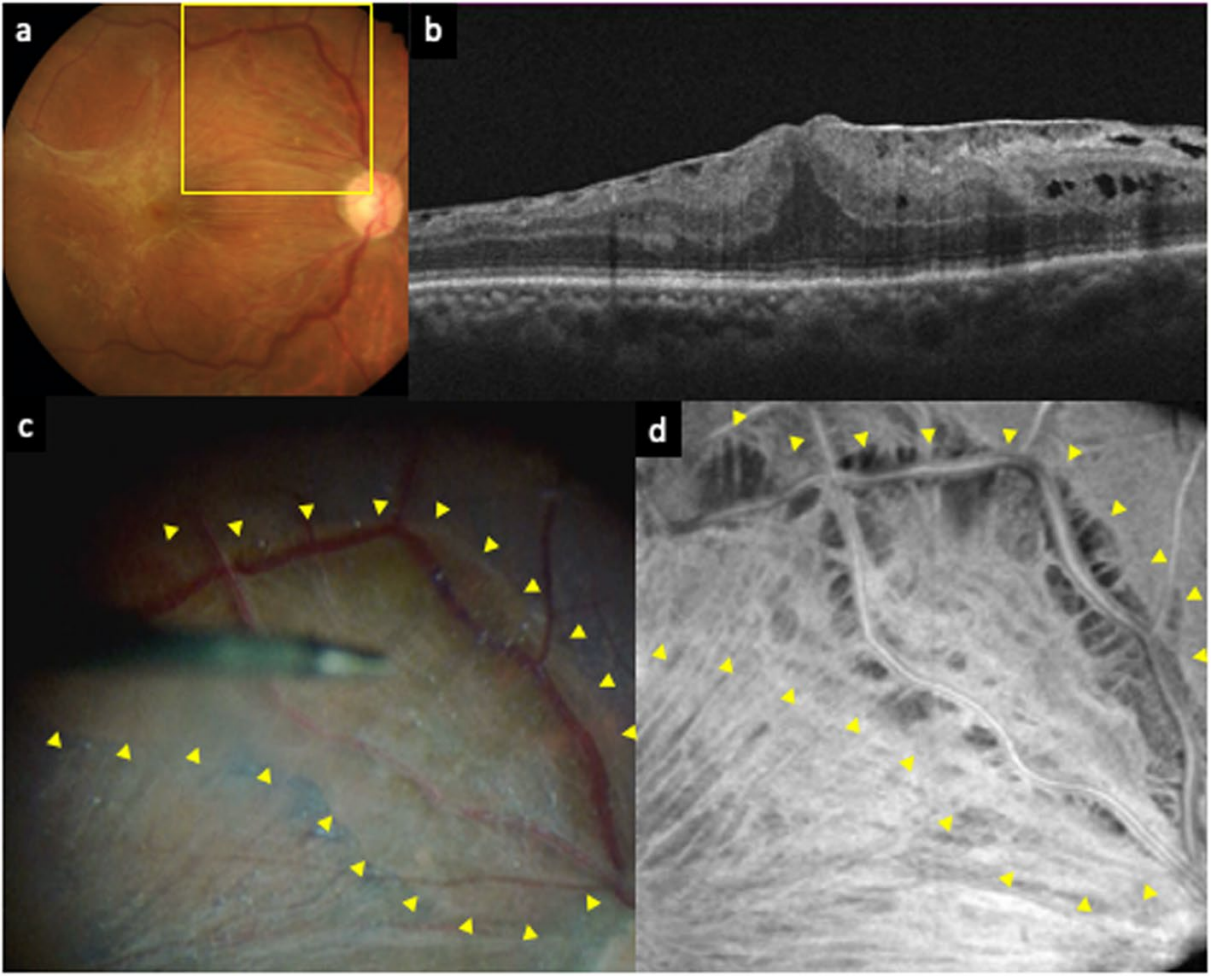
A preoperative fundus photography (**a**) of the right eye shows the epiretinal membrane (ERM). Optical coherence tomography (OCT) (**b**) shows the ERM with edema. An intraoperative image (**c**) and a preoperative OCT en-face image (**d**) correspond to the yellow box in the fundus photography (**a**). The retinal surface was stained with Brilliant Blue G (BBG) before peeling both the ERM and internal limiting membrane (ILM). The area surrounded by yellow arrowheads is not stained with BBG. A preoperative OCT en-face image (**d**) demonstrated dissociated optic nerve fiber layer-like appearance in the area where BBG was not stained (surrounded by yellow arrowheads in **c** and **d**)

A 67-year-old man presented with metamorphopsia and decreased vision in his right eye. The best-corrected visual acuity (BCVA) was 20/100, and slit-lamp examination revealed a pterygium and a moderate nuclear cataract. An idiopathic ERM was detected on fundus photography and OCT (Fig. 1a and b). The patient underwent simultaneous phacoemulsification, intraocular lens (IOL) implantation and PPV with ERM and ILM peeling in his right eye.

In the operation, after phacoemulsification, core vitrectomy was performed. The posterior vitreous detachment (PVD) had already occurred, which was confirmed by spraying triamcinolone acetonide during core vitrectomy. BBG was sprayed before the ERM and ILM peeling and the surgeon waited about 1 min with the BBG covering the macula. When the surgeon removed the BBG, an unstained area was identified (Fig. 1c). The surgeon carefully checked the area, which appeared to not be covered by an ERM. Therefore, the surgeon confirmed the absence of an ERM by starting the peeling from the opposite side of the unstained area and carefully extending toward it. The absence of an ERM at the area was verified and subsequent ILM peeling ended at the edge of the area. Only minute fiber-like tissues were observed at the surface of the lesion. The surgeon sprayed BBG again and reconfirmed that the area was not stained with BBG. Therefore, we diagnosed this lesion as an ILM defect. We carefully reviewed the preoperative OCT images acquired using RTVue XR Avanti (Optovue, Inc., Fremont, CA, USA) and noticed DONFL-like low-brightness spots in the ILM defect area on the OCT en-face image (Fig. 1d). Follow-up was performed up to 1 year postoperatively. The visual acuity improved to 20/20 and no complications were observed during these follow-up periods.

### Case 2

**Fig. 2:**
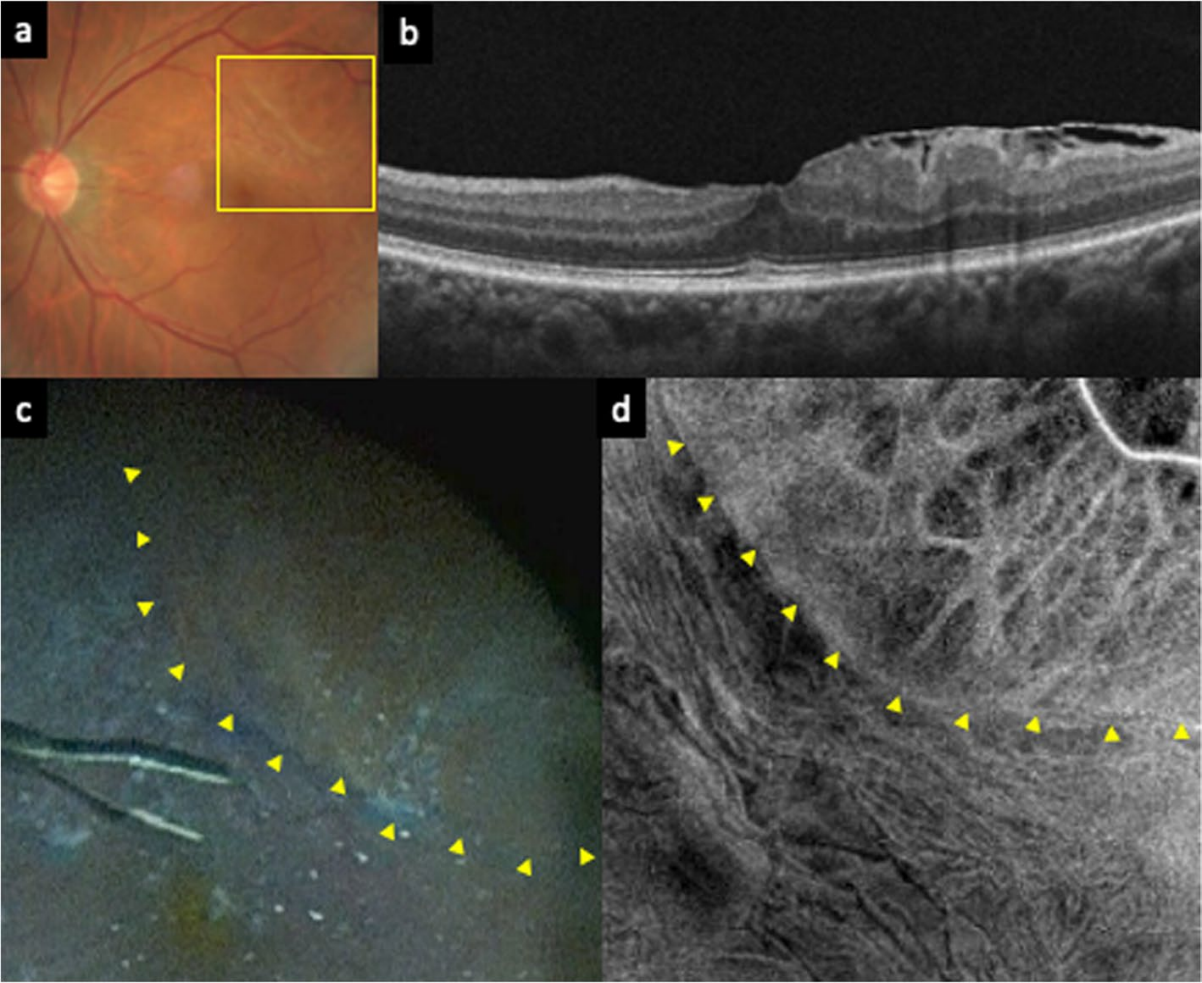
A preoperative fundus photograph (**a**) of the left eye shows the epiretinal membrane (ERM). Optical coherence tomography (OCT) (**b**) shows the ERM. An intraoperative image (**c**) and a preoperative OCT en-face image (**d**) correspond to the yellow box in the fundus photography (**a**). The retinal surface was stained with Brilliant Blue G (BBG) before peeling both the ERM and internal limiting membrane (ILM). The area surrounded by yellow arrowheads is not stained with BBG. A preoperative OCT en-face image (**d**) demonstrated dissociated optic nerve fiber layer-like appearance in the area where BBG was not stained (the border is indicated by yellow arrowheads in **c** and **d**)

A 73-year-old man presented with metamorphopsia and decreased vision in his left eye. The BCVA was 20/25, and slit-lamp examination revealed a moderate nuclear cataract. An idiopathic ERM was detected on fundus photography and OCT (Fig. 2a and b). The patient underwent simultaneous phacoemulsification, IOL implantation, and PPV with ERM and ILM peeling in his left eye.

Similar to case 1, the PVD had already occurred and an area that was unstained by BBG before ERM and ILM peeling was found (Fig. 2c). The absence of an ERM at the area was verified by peeling the ERM toward the unstained area from the opposite side, and the absence of an ILM at the area was verified by the fact that subsequent ILM peeling ended at the edge of the area. Only minute fibrin-like fibers were observed at the surface of the lesion. The surgeon sprayed BBG again and reconfirmed that the area was not stained with BBG. Therefore, we diagnosed that area as an ILM defect. We examined the OCT images acquired using Cirrus SD-OCT 5000 (Carl Zeiss Meditec Inc., Dublin, CA, USA) and noticed DONFL-like low-brightness spots in the area of the ILM defect on the OCT en-face images (Fig. 2d). Follow-up was performed up to 1 year postoperatively. The visual acuity improved to 20/20 and no complications occurred during the follow-up period.

## Discussion and conclusions

We described two cases in which areas with DONFL-like spots were detected on the OCT en-face images, and an ILM defect in those areas was confirmed during the ERM surgery. To the best of our knowledge, this is the first report to predict the 2-dimensional extension of an ILM defect by detecting an area with DONFL-like spots in the preoperative OCT en-face images.

Previous studies have reported that tearing and folding of the ILM associated with an ERM occur in 8.6 to 12.03% of cases [7, 8]. Moreover, Hussnain et al. described schisis of the RNFL (sRNFL) and a similar ILM defect, which they referred to as “ILM dehiscence” [9]. They reported that sRNFL occurred in 51.2% and an ILM defect was found in 76.2% of the cases with a sRNFL [9]. Therefore, an ILM defect may be a relatively common finding in ERM cases.

In the current cases, a DONFL-like low-brightness spots area was observed in the area of the ILM defect. In the previous reports, the edge of the ILM defect was observed as a hyporeflective band on near-infrared fundus images and the prominent ILM findings were referred to as a “spaghetti sign” on OCT B-scans [7, 9]. It is important to check for an ILM defect before surgery by evaluating the near-infrared fundus images, OCT B-scans, and en-face images. Moreover, en-face images may show all areas of the ILM defect.

A preoperative ILM defect is a potential pathogenesis of an ERM. Foos hypothesized that an initial process of ERM formation might be glial cell migration from the retina by microbreaks in the ILM [10–12]. The histopathologic studies demonstrated the proliferative changes on the retinal surface involving activated glial cells referred to as epivascular glia (EVG) [13, 14]. A recent study with high-resolution OCT also reported that ERMs were commonly observed in eyes with EVG [15]. Therefore, the preoperative ILM defects caused by contractile ERMs also may lead to worsening of ERMs.

Tadayoni et al. first noticed the unusual appearance of the optic nerve fiber layer after membrane peeling and referred to this appearance as the “DONFL” [16]. The DONFL is commonly observed after vitrectomy with ILM peeling and thought to be caused by ILM peeling. However, the DONFL can be observed even when the ILM is defective due to a disease in which ILM peeling is not performed [17, 18]. Therefore, as in those cases, the DONFL can be observed even if the ILM is defective due to an ERM.

The DONFL has been reported to occur in only 54 to 62% of patients who underwent ILM peeling [19, 20]. Considering that the DONFL is not observed in all cases in which the ILM is peeled, it is unknown if the DONFL is observed preoperatively in all areas with an ILM defect, so further research is needed.

In conclusion, we present two cases in which areas with DONFL-like spots were detected on the preoperative OCT en-face images, and defects in the ILM in those areas were confirmed during the ERM surgery. To avoid iatrogenic damage to the RNFL by touching/pinching it with forceps, predicting an ILM defect by detecting areas with DONFL-like spots in the preoperative OCT en-face images may be useful.

## Data Availability

Not applicable.

## Abbreviations

BBG: Brilliant Blue G
BCVA: Best-corrected visual acuity
DONFL: Dissociated optic nerve fiber layer
ERM: Epiretinal membrane
EVG: Epivascular glia
ILM: Internal limiting membrane
IOL: Intraocular lens
OCT: Optical coherence tomography
PPV: Pars plana vitrectomy
PVD: Posterior vitreous detachment
RNFL: Retinal nerve fiber layer
sRNFL: Schisis of the RNFL
